# Breaking the PRRSV-2 Life Cycle in Porcine Alveolar Macrophages: Tylvalosin’s Multi-Stage Inhibition

**DOI:** 10.3390/vetsci12040348

**Published:** 2025-04-09

**Authors:** Hui An, Yuhan Zhao, Xiaohong Deng, Wei Hu, Xia Zhang, Shuo Zheng, Longshuai Yao, Fanliang Meng, Zheng Fang, Fanghua Xu, Jianhua Qiu, Ning Li, Gang Wang

**Affiliations:** 1 Shandong Provincial Key Laboratory of Zoonoses, College of Veterinary Medicine, Shandong Agricultural University, Taian 271018, China; ah15296611405@outlook.com (H.A.); zhaoyh2023@163.com (Y.Z.); 17865681152@163.com (X.Z.); zs1012635836@163.com (S.Z.); a956278914@163.com (L.Y.); 18754875921@163.com (F.M.); 17864809007@163.com (Z.F.); a1600501451@126.com (F.X.); qiujianhua@sdau.edu.cn (J.Q.); 2 Zhejiang ECO-BIOK Animal Health, Shanghai 200063, China; xh.deng@eco-biok.com; 3 College of Veterinary Medicine, Xichang University, Xichang 415000, China; 4 College of Veterinary Medicine, Gansu Agricultural University, Lanzhou 730070, China; huwei@eco-biok.com

**Keywords:** porcine reproductive and respiratory syndrome virus, tylvalosin, antiviral activity, viral replication cycle

## Abstract

Porcine reproductive and respiratory syndrome (PRRS) is one of the most serious diseases threatening the swine industry worldwide. However, no satisfactory control strategy has existed until now. In this study, the effectiveness of tylvalosin against PRRSV and the underlying mechanism was investigated. The results showed that in porcine alveolar macrophages (PAMs), tylvalosin can inhibit the replication of the NADC30-like and NADC34-like strains in a dose-dependent manner. It is worth noting that pre-incubation with tylvalosin had no significant inhibitory effect on the NADC30-like strain but did inhibit the NADC34-like strain. Co-incubation of both viruses and tylvalosin or post-incubation with tylvalosin after viral infection inhibited PRRSV. We further analyzed the effect of tylvalosin on different stages of PRRSV replication and found that the stages in the PRRSV life cycle could be blocked by tylvalosin. Tylvalosin has an antiviral effect on all four stages of the NADC34-like strain’s infectious cycle but has no effect against the adsorption phase of the NADC30-like strain. These results demonstrated that tylvalosin suppressed PRRSV infection in PAMs and inhibited PRRSV infection at multiple steps of the viral life cycle. This study will contribute to the clinical prevention and control of PRRS and provide a basis for further exploration of the anti-PRRSV effects of tylvalosin.

## Simple Summary

Porcine reproductive and respiratory syndrome (PRRS) remains a significant threat to the global swine industry, with no fully effective control measures currently available. This study explored the efficacy of tylvalosin against PRRSV and its potential mechanisms of action. The findings indicated that tylvalosin effectively inhibited the replication of both NADC30-like and NADC34-like strains of PRRSV in porcine alveolar macrophages (PAMs), with the inhibitory effect increasing as the concentration of tylvalosin rose. Interestingly, pre-treatment with tylvalosin did not significantly impact the replication of the NADC30-like strain but did suppress the NADC34-like strain. However, when tylvalosin was co-incubated with the viruses or administered post-infection, it successfully inhibited PRRSV replication. Further investigation revealed that tylvalosin could interfere with multiple stages of the PRRSV life cycle. Specifically, it demonstrated antiviral activity against all four stages of the NADC34-like strain’s infectious cycle but did not affect the adsorption phase of the NADC30-like strain. Overall, these results highlight tylvalosin’s ability to suppress PRRSV infection in PAMs by targeting various stages of the viral life cycle. This research not only contributes to the clinical management of PRRS but also provides a foundation for future studies on the antiviral potential of tylvalosin against PRRSV.

## 1. Introduction

Porcine reproductive and respiratory syndrome (PRRS) is an acute infectious disease caused by porcine reproductive and respiratory syndrome virus (PRRSV); PRRS is characterized by porcine reproductive and respiratory disorders and is commonly known as “blue ear disease” [1]. PRRSV can infect pigs at different stages of growth, causing abortion, stillbirth, and mummified fetuses in pregnant sows; respiratory diseases in piglets and growing pigs; and immunosuppression and increased risk of secondary infection. This disease has resulted in a large negative economic impact on the global pig industry [2].

The first outbreak of PRRS was reported in China in 1995, and a highly pathogenic variant of PRRSV (HP-PRRSV), represented by the strain JXA1, emerged in 2006, causing devastation to the pig industry in China [3,4,5,6]. PRRSV has two main genotypes: PRRSV-1 and PRRSV-2. In China, at present, the prevalent PRRSV strains belong to genotype 2. Due to continuous mutation and genetic recombination of PRRSV, the PRRSV strains are increasingly complex, and according to the viral open reading frame 5 (ORF5) sequence, PRRSV-2 can be divided into nine lineages [7], with the prevalent strains of PRRSV in China mainly belonging to lineage 1 (NADC30-like and NADC34-like), lineage 8 (highly pathogenic strains), lineage 3 (recombinant strains), and lineage 5 (classical strains) [8]. Since the introduction of the NADC30 strain in China, its epidemic scope has been expanding, and by 2024, the NADC30 strain replaced the dominant epidemic position of the traditional HP-PRRSV strain. The prevalence of the HP-PRRSV-like strain is second only to the NADC30-like strain, and the prevalence of the NADC34lLike strain is increasing day by day [9]. Compared with HP-PRRSV, the virulence of the NADC30 strains is relatively low but their genetic variability and genetic recombination are extremely extensive, and most of them produce genetic recombination with different lineage strains, resulting in the generation of new strains with different virulence, which not only increases the diversity of PRRSV epidemic strains in China but also increases the difficulty of epidemic prevention and control. Additionally, the positive rate of PRRSV in most areas is as high as more than half [10,11].

Vaccination is currently a widely used method for preventing PRRSV infection. There are attenuated, inactivated, and genetically engineered vaccines against PRRSV. However, because of the mutation and recombination of the virus, as well as other factors such as the efficacy and safety of the vaccine itself, commercial vaccines cannot provide satisfactory protection [12,13,14,15]. Therefore, the development of new anti-PRRSV strategies is important for PPRS prevention and control. Antiviral drugs have attracted increasing attention. Antibiotics, herbal extracts, and nano-antibodies have also shown anti-PRRSV effects. For example, glycyrrhiza polysaccharide, an active component extracted from glycyrrhiza can inhibit PRRSV replication in a dose-dependent manner [16]. Ivermectin treatment has also inhibited PRRSV infection in PAM-pCD163 cells [17].

Tylvalosin is the third generation of a macrolide antibiotic that is a derivative of Tylosin. It has lower toxicity and can rapidly concentrate in various host cells, especially in primary porcine alveolar macrophages (PAMs), which are target cells of PRRSV [18]. Tylvalosin, like other macrolide antibiotics, interferes with protein synthesis by binding to the 50S ribosomal subunit. Tylvalosin has antibacterial, anti-mycoplasmic, anti-helminthic, anti-inflammatory, and immunomodulatory effects, and it can increase macrophage phagocytic activity as well as reduce oxidative stress and the expression of pro-inflammatory cytokines [19]. It has been shown that administration via feed reduced the stress responses of pigs during immunization with an inactivated PRRSV vaccine [20]. Tylvalosin is often used for the treatment and control of porcine typhoid pneumonia, proliferative enteritis, mycoplasma pneumoniae, and Pasteurella multocida infection [21]. In addition, tylvalosin also has antiviral activities; it was found that tylvalosin inhibited the replication of PRRSV and increased the antibody levels against PRRSV [22,23,24].

The growing challenge of PRRSV prevention caused by its mutation and recombination poses a great threat to the swine industry. Tylvalosin has a broad antimicrobial spectrum, good antimicrobial activity, a low cost, and is widely used in animal husbandry worldwide. Therefore, further studies on the inhibitory effect and underlying mechanism of tylvalosin on different PRRSV strains are necessary. In this study, the effect of tylvalosin on the different PRRSV strains was determined.

## 2. Materials and Methods

### 2.1. Cells and Viruses

Porcine alveolar macrophages (PAMs), NADC30-like PRRSV (SDVD-JNJX), and NADC34-like PRRSV (MDC) were preserved in our laboratory. PAMs were cultured in RPMI 1640 supplemented with 10% fetal bovine serum (FBS) and 1% penicillin, streptomycin, and amphotericin B.

### 2.2. Reagent and Antibody Suppliers

HiScript II RT SuperMix for qPCR was purchased from Vazyme (Nanjing, China). Penicillin–streptomycin–amphotericin B and skimmed milk powder were purchased from Solarbao (Beijing, China). RPMI 1640 medium and PBS were obtained from Gibco (Dalian, China). FBS came from Vazyme (Nanjing, China). Tylvalosin (825.5 ug/mg) was provided by Zhejiang ECO-BIOK Animal Health Products Co., Ltd. (Shanghai, China). Anti-β-actin antibody and HRP-labeled goat anti-mouse IgG were purchased from Beyotime (Shanghai, China). FITC-labeled goat anti-mouse IgG was bought from ZSGB-BIO (Beijing, China).

### 2.3. Cell Viability Assay

A Cell Counting Kit-8 (CCK-8) (Proteintech, Wuhan, China) was used to detect the cytotoxicity of tylvalosin in PAMs. PAMs were cultured in 96-well plates followed by incubation with different concentrations of tylvalosin (0, 1, 10, 25, and 50 μg/mL) for 36 h post-inoculation (hpi). Then, 10 µL of CCK-8 reagent was added to each well and incubated for 2 h at 37 °C. The optical density (OD) value of the wells at a wavelength of 450 nm was determined using a microplate reader (BIO-RAD, Beijing, China). Cell activity (%) = [OD experimental wells − OD blank wells]/[OD control wells − OD blank wells] × 100%.

### 2.4. Assay for Testing the Inhibition of PRRSV Replication

Three different treatments were used to explore the inhibitory effect of tylvalosin on NADC30-like and NADC34-like PRRSV strains.

First, in the pre-treatment group test with tylvalosin, PAMs were incubated in 6-well plates and treated with various concentrations of tylvalosin (10, 15, 20, 25, and 30 μg/mL) for 4 h. Then, the cells were inoculated with NADC30-like or NADC34-like strains with multiplicity of infection (MOI) at 0.2 and incubated at 37 °C in a 5% CO_2_ incubator. After 2 h, RPMI 1640 containing 2% FBS was placed in the incubator and cells continued to be cultured for 36 h.

Next, tests of the co- and post-inoculation treatment groups were also performed. In the co-treatment test with tylvalosin and PRRSV, PAMs were incubated with different concentrations of tylvalosin (10, 15, 20, 25, and 30 μg/mL) and the virus for 2 h. Then, the 2% FBS containing different concentrations of tylvalosin was changed and cells continued to be incubated for 36 h. In the post-treatment test, the cells were first inoculated with PRRSV and then tylvalosin was added. The cells were incubated with PRRSV for 2 h, followed by washing three times with PBS. Then, 2% FBS 1640 containing different concentrations of tylvalosin was added to the culture cells for 36 h. Finally, the cells were collected for quantitative real-time PCR (qPCR) and Western blot analyses.

### 2.5. Effect of Tylvalosin on the Life Cycle of PRRSV

To investigate the effect of tylvalosin on PRRSV adsorption, the PAMs were first incubated at 4 °C for 1 h, and then co-incubated with different concentrations (10, 15, 20, 25, or 30 μg/mL) of tylvalosin and the viruses (MOI = 0.2) at 4 °C for 4 h. Unbound viruses were washed away with PBS. The medium was replaced with 2% FBS 1640 and continued to be incubated at 37 °C with 5% CO_2_.

Next, to investigate the effect of tylvalosin on PRRSV penetration and uncoating, viruses were used to pre-infect the cells at 4 °C for 4 h. The cells were washed three times with PBS, and the maintenance medium containing the different concentrations of tylvalosin (10, 15, 20, 25, or 30 μg/mL) was replaced. After 6 h, the medium was replaced by 2% FBS 1640 for further incubation.

Lastly, to examine replication and synthesis or the assembly and budding of PRRSV, viruses were used to pre-infect the cells at 37 °C for 6 h or 24 h. Cells were washed three times with PBS, and the medium was incubated for 6 h with 2% FBS 1640 containing the different concentrations of tylvalosin. Then, the medium was replaced for further incubation. The cells continued to be cultured for 36 h for qPCR and Western blot analyses.

### 2.6. Immunofluorescence Assay (IFA)

PAMs were seeded in 24-well plates (1 × 10^6^ cells/well), and the cells were treated with the three different treatments, as described above. The virus was observed at 36 hpi using the IFA method. In brief, the cells were fixed with 4% paraformaldehyde at room temperature for 20 min and permeabilized with 0.1% TritonX-100 at 37 °C for 15 min. After blocking with 5% bovine serum albumin for 30 min at 37 °C, PRRSV was detected with a mouse monoclonal antibody against the N protein (1:1000) and FITC-labeled goat anti-mouse secondary antibody (1:500). After washing three times with PBS, the cell staining was visualized using an inverted fluorescence microscope (Nikon, Tokyo, Japan).

### 2.7. Quantitative Real-Time PCR

The total RNA of the cell samples was extracted using the Ultrapure RNA Kit (CWBIO, Taizhou, China). Then, RNA was used for cDNA synthesis using PrimeScript RT Master Mix (Takara, Dalian, China). The primers and probes used for qPCR are listed in Table 1. The thermal cycling parameters were as follows: 40 cycles at 94 °C for 30 s, 94 °C for 5 s, and 60 °C for 34 s. All samples were tested in triplicate on the same plate.

### 2.8. Western Blot Analysis

The cell samples were lysed in RIPA lysis buffer (NCM, Suzhou, China) containing 1% protease inhibitors (Beyotime, Shanghai, China). The supernatant was collected after centrifugation, determined by the BCA protein assay kit (Beyotime, Shanghai, China), and then boiled with 5× sample loading buffer. The equivalent volume of protein samples was separated using 12.5% SDS-PAGE gels and transferred to PVDF membranes. After membrane transfer, the membranes were closed with 5% skimmed milk powder at room temperature for 2 h. After that, the membrane was incubated at 4 °C overnight with PRRSV N protein monoclonal antibody, with β-actin serving as control. Then, the membranes were incubated with secondary antibodies containing HRP-labeled goat anti-mouse for 2 h at room temperature. The blots were detected using enhanced chemiluminescent (ECL) reagent (Fdbio, Science, Hangzhou, China).

### 2.9. Statistical Analysis

All data were presented as the mean ± standard deviation (SD) of at least three independent experiments. The data were analyzed using GraphPad Prism 8.0 (GraphPad, San Diego, CA, USA). Differences compared to the control group are indicated by * *p <* 0.05, ** *p <* 0.01, and *** *p <* 0.001. *p* values less than 0.05 were considered to be statistically significant.

## 3. Results

### 3.1. Cytotoxicity Test of Tylvalosin in PAMs

**Figure 1:**
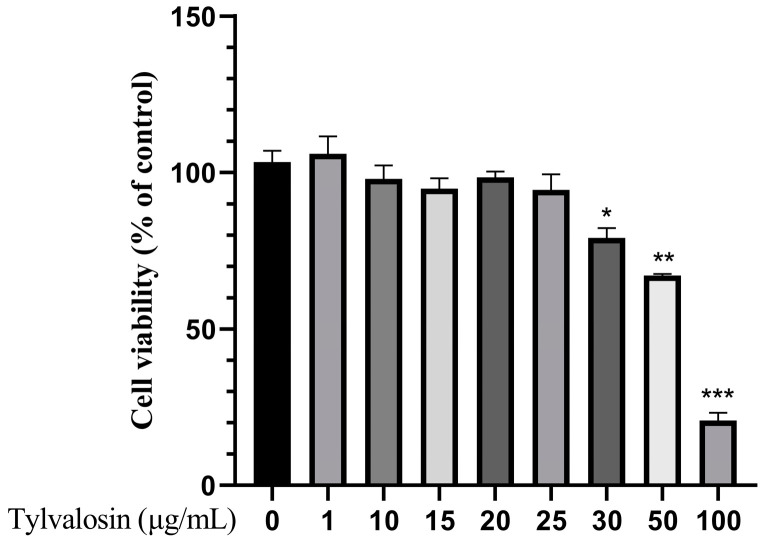
Result of tylvalosin cytotoxicity test in PAMs. Cells were treated with various concentrationsof tylvalosin (1, 10, 15, 20, 25, 30, 50, 100 ug/mL) for 36 h. Relative cell viability was determined by CCK-8. The differences were represented by * (*p* < 0.05), ** (*p* < 0.01), *** (*p* < 0.001).

To validate the effect of tylvalosin on PRRSV replication in PAMs, the toxicity of tylvalosin on PAMs was first studied. As shown in Figure 1, the cell activity of the normal growing cells was regarded as 100%, and there was no significant effect of tylvalosin on the cell activity until its concentration reached 50 μg/mL. Meanwhile, cell activity was also inhibited significantly (*p* < 0.001). Therefore, tylvalosin has no toxic effect on PAMs at concentrations of 25 μg/mL or less.

### 3.2. Tylvalosin Inhibits PRRSV Replication In Vitro

**Figure 2:**
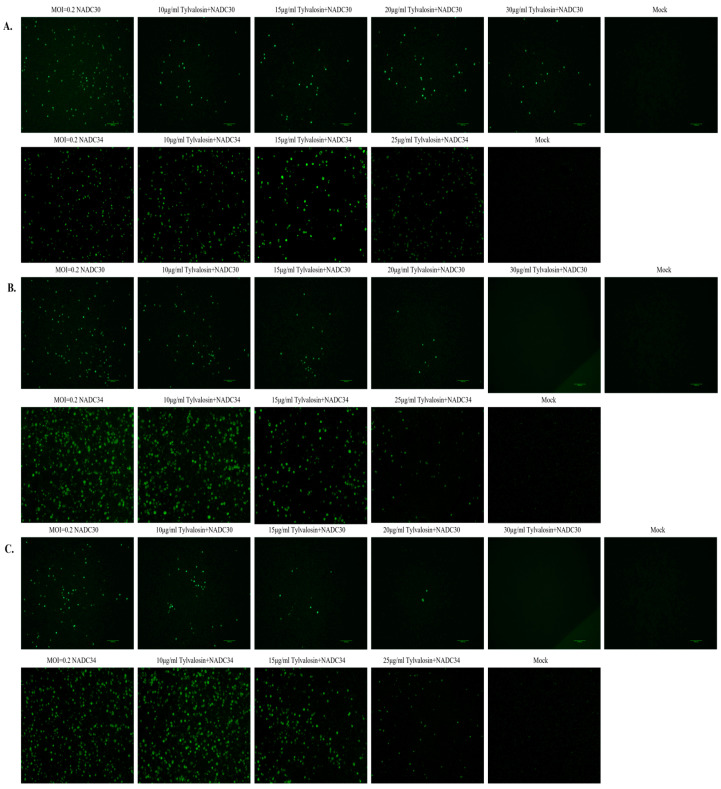
Inhibition of PRRSV proliferation assay by tylvalosin via IFA. (**A**) In the pre-treatment group, cells were incubated with tylvalosin for 4 h in advance. (**B**) In the co-treatment group, tylvalosin was incubated with the virus for 2 h and replaced with 2% FBS, containing different concentrations of tylvalosin. (**C**) In the post-treatment group, cells were first infected with the virus and then incubated with different concentrations of tylvalosin. Cells were collected at 36 h for IFA analysis.

Three different treatments were used to study the inhibitory effect of tylvalosin on NADC30-like and NADC34-like strains on PAMs. At 36 hpi, the immunofluorescence assay (IFA) was carried out to detect the proliferation of PRRSV. As shown in Figure 2A, pre-treatment with tylvalosin was not effective against the NADC30-like strain but it did inhibit the NADC34-like strain to some extent. However, in the co-treatment and post-treatment groups, the results showed that PRRSV replication in cells gradually decreased with increasing concentrations of tylvalosin (Figure 2B,C), indicating that tylvalosin inhibited PRRSV replication in a dose-dependent manner.

**Figure 3:**
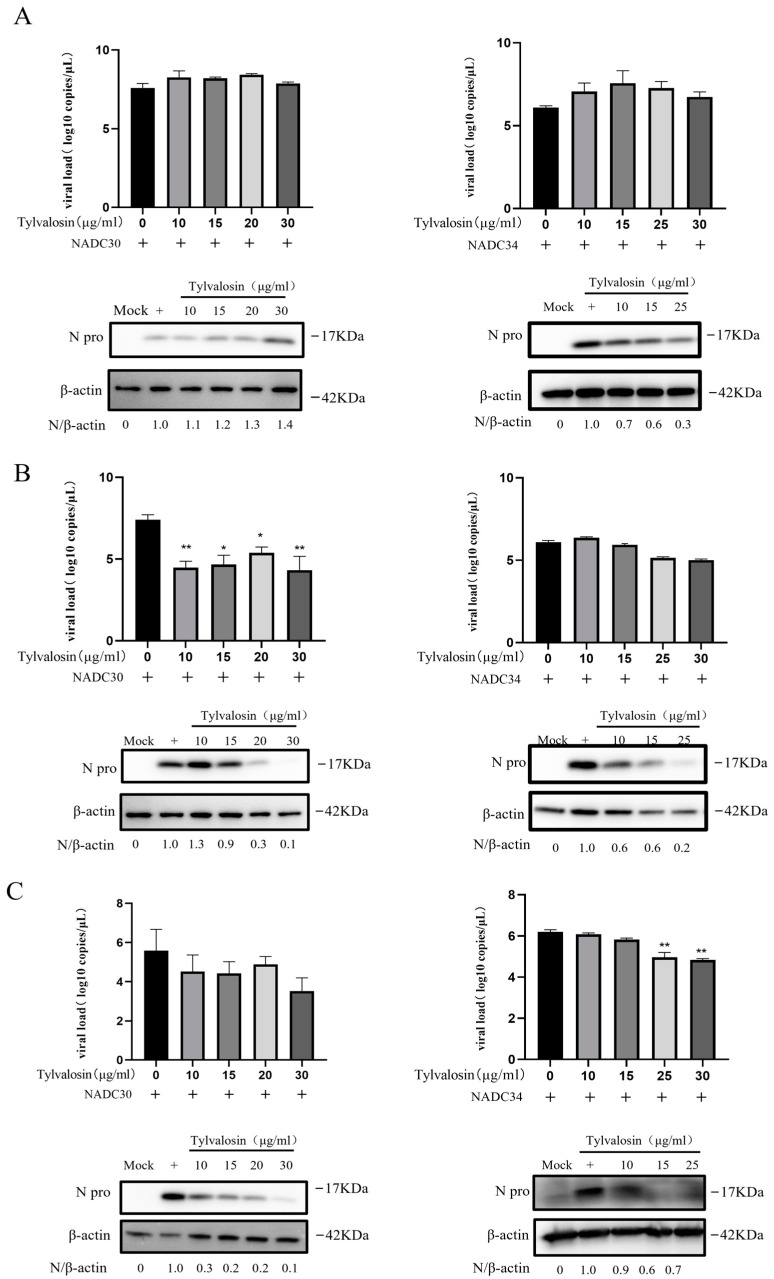
PRRSV N protein expression level and virus copy number in PAMs with different treatments of tylvalosin (original Western blot figures see Supplementary Materials). (**A**) Pre-treatment of tylvalosin groups. The cells were incubated for 4 h with tylvalosin and then infected with the virus. (**B**) Co-treatment groups. Tylvalosin was co-incubated with the virus for 2 h and then replaced with fresh 2% FBS of tylvalosin. (**C**) Post-treatment of tylvalosin groups. Cells were infected with the virus and then incubated with tylvalosin. The protein bands were analyzed by grayscale analysis of Image J (version 1.8) software to calculate the ratio of the N protein to the internal reference protein β-actin. The data were shown as means ± SD (*n* = 3). Asterisks indicate a statistically significant difference compared with untreated cells (* *p* < 0.05, ** *p* < 0.01).

To further validate the anti-PRRSV effect of tylvalosin, we examined the expression of N proteins and viral copy numbers in the different groups. As shown in Figure 3A, pre-treatment with tylvalosin had no significant inhibitory effect on the NADC30-like strain but it did inhibit the NADC34-like strain at the protein level. Similarly, the co- and post-treatment groups showed that the expressions of viral N proteins and mRNA gradually decreased in infected cells as the concentrations of tylvalosin increased (Figure 3B,C).

### 3.3. Tylvalosin Inhibits Multiple Stages of PRRSV Replication

**Figure 4:**
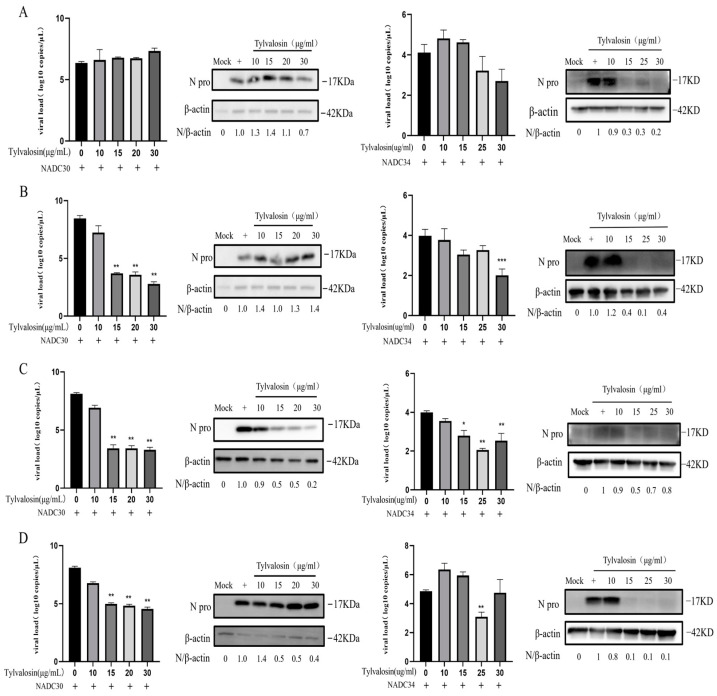
The effect of tylvalosin on the life cycle of PRRSV (original Western blot figures see Supplementary Materials). (**A**) The effect of tylvalosin at the attachment stage of the NADC30-like and NADC34-like strains on PAMs. Cells were placed at 4 °C for 1 h, and then the virus, cells, and tylvalosin were co-incubated at 4 °C for 4 h. (**B**) The effect of tylvalosin on the viral penetration and uncoating stage. Viruses infected cells at 4 °C for 4 h, followed by incubation with tylvalosin for 6 h. (**C**) The effect of tylvalosin on the replication stage of the PRRSV in PAMs. Viruses infected cells at 37 °C for 4 h, followed by incubation with tylvalosin for 6 h. (**D**) The effect of tylvalosin on the assembly and budding stages of PRRSV. Viruses infected cells at 37 °C for 24 h, followed by incubation with tylvalosin. The data were shown as means ± SD (*n* = 3). Asterisks indicate a statistically significant difference compared with untreated cells (* *p* < 0.05; ** *p* < 0.01).

To further explore the effect of tylvalosin on the PRRSV life cycle (adsorption, penetration and uncapping, replication and synthesis, and assembly and budding phases), PAMs were incubated with tylvalosin at different stages of the PRRSV replication cycle. The effect of tylvalosin on viral adsorption was first examined. The results showed that tylvalosin reduced the NADC34-like strain mRNA expression and N protein production (Figure 4A). However, tylvalosin did not inhibit the adsorption of the NADC30-like strain. As shown in Figure 4B, virus replication was also significantly inhibited when the infected cells were treated with tylvalosin during penetration and decapsulation. In addition, tylvalosin inhibited the replication and synthesis, as well as the assembly and budding stages, of both NADC30-like and NADC34-like strains (Figure 4C,D). These results indicated that tylvalosin can inhibit PRRSV replication by targeting the multiple stages of the virus life cycle.

## 4. Discussion

PRRSV is still one of the most virulent pathogens in global pig production, causing huge economic losses to the pig industry. The emergence of NADC30-like and NADC34-like strains has led to their becoming the predominant endemic strains and threatening the pig production system in China [25,26,27]. Vaccination is the main strategy to prevent and control PRRS, but PRRS vaccines do not have strong cross-protection against heterologous strains, resulting in still high PRRS-positive rates on farms. It is therefore necessary to develop new prevention and control strategies, especially by conducting research on effective vaccines and specific drugs to cope with mutant strains of PRRSV.

Tylvalosin is a broad-spectrum veterinary macrolide antibiotic and is currently widely used on pig farms to control respiratory and enteric bacterial pathogens. Recent studies have shown that tylvalosin can act as an anti-PRRSV drug and has the property of non-specifically inhibiting PRRSV proliferation both in vitro and in vivo [19]. Tylvalosin effectively inhibits the proliferation of PRRSV, and can also be found in MARC-145 cells [28,29]. MARC-145 cells are commonly used to explore the PRRSV replication cycle but the target cells of PRRSV are PAMs, and the results obtained from antiviral assays performed on target cells are more clinically representative. The safe concentration of tylvalosin has been reported to vary from 40 µg/mL in MARC-145 cells to 20 or 40 µg/mL in PAMs, depending on the quality of the products produced by different manufacturers [28]. In this study, PAMs were used to evaluate the anti-PRRSV efficacy of tylvalosin. We treated PAMs with tylvalosin using three different methods (pre-, co-, and post-treatment) and analyzed the effect of PRRSV inhibition by tylvalosin. The results showed that the co-treatment and post-treatment groups of tylvalosin significantly inhibited viral replication and that the effective concentration in PAMs was more than 15 μg/mL. It is worth noting that tylvalosin incubation before virus inoculation had no inhibitory effect on the NADC30-like strain but it did inhibit the NADC34-like strain to some extent. In this study, the results are inconsistent with those previously obtained from the NADC30-like strain in MARC-145 cells [29]. PAM cells, susceptible to PRRSV in pigs, are more sensitive to PRRSV infection, and therefore, the situation presented is also more obvious than that in MARC-145 cells. Both strains belong to lineage 1, but the NADC30-like strain has a 31 amino acid deletion in the NSP2 sequence compared to the NADC34-like strain. Studies have shown that the NADC34 strain has a stronger replication ability than the NADC30 strain and weaker pathogenicity [30]. The genetic and virulence differences between the two strains are one of the important factors for the differences in the experimental results.

The virus replication cycle consists of four phases: adsorption, penetration and uncoating, protein synthesis, and assembly and budding. Disruption of any one of these processes can affect viral replication. During PRRSV replication, it first needs to enter the host cell through the endocytosis mediated by the receptor on the cell membrane. It then fuses with the endosomal membrane to release nucleic acids into the cytoplasm, then completes replication. We designed a time course test to determine the stage in which tylvalosin exhibits anti-PRRSV activity. The results showed that tylvalosin had an inhibitory effect on all four stages of the replication cycle of the NADC34-like strain but it did not have a significant effect on the adsorption of the NADC30-like strain. The polyphenolic compound EGCG extracted from green tea acts as an antiviral receptor inhibition of PRRSV replication in MARC-145 cells by attaching to the active site of the PRRSV glycoprotein (GP5) and partially competing for viral particle binding with heparin and salivary acid [31]. We speculated that the effect of tylvalosin on adsorption may be related to the virulence of the strain and the ability of the virus to bind to the cellular receptor, suggesting that tylvalosin may interfere with the binding of the strain to the receptor on the surface of the cell.

PRRSV infections require a low pH step in endosomes to accomplish their fusion with endosomal membranes. Tylvalosin was reported to be able to raise endosomal pH, and this may be an important mechanism by which it inhibits PRRSV replication. In addition, some herbal medicines such as curcumin reduce PRRSV replication by preventing PRRSV internalization and PRRSV-mediated cell fusion in MARC-145 cells and PAMs [18]. In addition, some herbal medicines such as curcumin reduce PRRSV replication by preventing PRRSV internalization and PRRSV-mediated cell fusion in MARC-145 cells and PAMs [32]. Spiramycin and azithromycin, which are macrolide antibiotics like tylvalosin [33], possess antiviral activities against enterovirus and coxsackievirus likely by interfering with viral RNA replication, which may also be responsible for the inhibition of PRRSV by tylvalosin at the replication and synthesis stages.

In addition to potentially directly targeting viral replication, tylvalosin can regulate the host signal transduction pathways through transcriptomics, such as protein digestion and absorption, PI3K-Akt signaling, FoxO signaling, and ferroptosis pathways, as well as up-regulating the expression of antiviral genes HMOX1, ATF3, and FTH1 in MARC-145 cells [28]. Animal studies have found that tylvalosin can attenuate IkBa phosphorylation and degradation and block NF-kB p65 translocation, thereby attenuating lung injury caused by PRRSV [19]. Therefore, effective modulation of host immune function may also be an important reason for the anti-PRRSV viral effects of tylvalosin.

## 5. Conclusions

In conclusion, our data demonstrated that tylvalosin inhibited PRRSV replication in PAMs by targeting multiple steps of the PRRSV replication cycle. Further studies are required to explore the precise antiviral mechanism for this effect.

## Figures and Tables

**Table 1:** Primer and probe sequences of RT-qPCR.

Name of Primer and Probe	Primer and Probe Sequence	Amplification Position (bp)	Target Gene	Amplification Size (bp)
NL-PRRSV-2-F	GGACGACAAATGCGTGGTT	14321–14339 14370–14388	ORF6	68
NL-PRRSV-2-R	CCACCACGTTGAAAGTGCC		ORF6	
NL-PRRSV-2-P	FAM-CTTGCCGTTATCGGATGA-MGB	14348–14365		

## Data Availability

The data presented in this study are available in the article.

## Supplementary Materials

The following supporting information can be downloaded at: https://www.mdpi.com/article/10.3390/vetsci12040348/s1.

## References

[1] Lunney J.K., Fang Y., Ladinig A., Chen N., Li Y., Rowland B., Renukaradhya G.J. (2016). Porcine Reproductive and Respiratory Syndrome Virus (PRRSV): Pathogenesis and Interaction with the Immune System. Annu. Rev. Anim. Biosci..

[2] Lunney J.K., Benfield D.A., Rowland R.R. (2010). Porcine reproductive and respiratory syndrome virus: An update on an emerging and re-emerging viral disease of swine. Virus Res..

[3] Guo Z., Chen X.X., Li R., Qiao S., Zhang G. (2018). The prevalent status and genetic diversity of porcine reproductive and respiratory syndrome virus in China: A molecular epidemiological perspective. Virol. J..

[4] Li Y., Wang X., Bo K., Wang X., Tang B., Yang B., Jiang W., Jiang P. (2007). Emergence of a highly pathogenic porcine reproductive and respiratory syndrome virus in the Mid-Eastern region of China. Vet. J..

[5] Tian K., Yu X., Zhao T., Feng Y., Cao Z., Wang C., Hu Y., Chen X., Hu D., Tian X. (2007). Emergence of fatal PRRSV variants: Unparalleled outbreaks of atypical PRRS in China and molecular dissection of the unique hallmark. PLoS ONE.

[6] Tong G.Z., Zhou Y.J., Hao X.F., Tian Z.J., An T.Q., Qiu H.J. (2007). Highly pathogenic porcine reproductive and respiratory syndrome, China. Emerg. Infect. Dis..

[7] Shi M., Lam T.T., Hon C.C., Murtaugh M.P., Davies P.R., Hui R.K., Li J., Wong L.T., Yip C.W., Jiang J.W. (2010). Phylogeny-based evolutionary, demographical, and geographical dissection of North American type 2 porcine reproductive and respiratory syndrome viruses. J. Virol..

[8] Han G., Lei K., Xu H., He F. (2020). Genetic characterization of a novel recombinant PRRSV2 from lineage 8, 1 and 3 in China with significant variation in replication efficiency and cytopathic effects. Transbound. Emerg. Dis..

[9] Zhao Y.Y., Ma X., Chen X.M., Song Y.P., Zheng L.L., Ma S.J., Chen H.Y. (2024). Molecular detection and genetic characteristics of porcine reproductive and respiratory syndrome virus in central China. Microb. Pathog..

[10] Huang B., Xu T., Luo Z., Deng L., Jian Z., Lai S., Ai Y., Zhou Y., Ge L., Xu Z. (2024). Prevalence and genetic diversity of PRRSV in Sichuan province of China from 2021 to 2023: Evidence of an ongoing epidemic transition. Virology.

[11] Zhou L., Han J., Yang H. (2024). The evolution and diversity of porcine reproductive and respiratory syndrome virus in China. Vet. Microbiol..

[12] Chai W., Liu Z., Sun Z., Su L., Zhang C., Huang L. (2020). Efficacy of two porcine reproductive and respiratory syndrome (PRRS) modified-live virus (MLV) vaccines against heterologous NADC30-like PRRS virus challenge. Vet. Microbiol..

[13] Sun Y.F., Zhou L., Bian T., Tian X.X., Ren W.K., Lu C., Zhang L., Li X.L., Cui M.S., Yang H.C. (2018). Efficacy evaluation of two commercial modified-live virus vaccines against a novel recombinant type 2 porcine reproductive and respiratory syndrome virus. Vet. Microbiol..

[14] Zhou L., Ge X., Yang H. (2021). Porcine Reproductive and Respiratory Syndrome Modified Live Virus Vaccine: A “Leaky” Vaccine with Debatable Efficacy and Safety. Vaccines.

[15] Zhou L., Yang B., Xu L., Jin H., Ge X., Guo X., Han J., Yang H. (2017). Efficacy evaluation of three modified-live virus vaccines against a strain of porcine reproductive and respiratory syndrome virus NADC30-like. Vet. Microbiol..

[16] Duan E., Wang D., Fang L., Ma J., Luo J., Chen H., Li K., Xiao S. (2015). Suppression of porcine reproductive and respiratory syndrome virus proliferation by glycyrrhizin. Antivir. Res..

[17] Lee Y.J., Lee C. (2016). Ivermectin inhibits porcine reproductive and respiratory syndrome virus in cultured porcine alveolar macrophages. Arch. Virol..

[18] Stuart A.D., Brown T.D.K., Mockett A.P.A. (2008). Tylvalosin, a macrolide antibiotic, nhibits the in-vitro replication of European and American porcine reproductive and respiratory syndrome virus (PRRS) viruses. Pig J..

[19] Zhao Z., Tang X., Zhao X., Zhang M., Zhang W., Hou S., Yuan W., Zhang H., Shi L., Jia H. (2014). Tylvalosin exhibits anti-inflammatory property and attenuates acute lung injury in different models possibly through suppression of NF-κB activation. Biochem. Pharmacol..

[20] Zhang D., Xia Q., Wu J., Liu D., Wang X., Niu Z. (2011). Construction and immunogenicity of DNA vaccines encoding fusion protein of murine complement C3d-p28 and GP5 gene of porcine reproductive and respiratory syndrome virus. Vaccine.

[21] Lopez Rodriguez A., Berge A.C., Ramage C., Saltzman R., Domangue R.J., Gnozzio M.J., Muller A., Sierra P., Benchaoui H.A. (2020). Evaluation of the clinical efficacy of a water soluble formulation of tylvalosin in the control of enzootic pneumonia associated with Mycoplasma hyopneumoniae and Pasteurella multocida in pigs. Porc. Health Manag..

[22] Arsic B., Barber J., Čikoš A., Mladenovic M., Stankovic N., Novak P. (2018). 16-membered macrolide antibiotics: A review. Int. J. Antimicrob. Agents.

[23] Du K., Xia Y., Wu Q., Yin M., Zhao H., Chen X.W. (2024). Analysis of whole transcriptome reveals the immune response to porcine reproductive and respiratory syndrome virus infection and tylvalosin tartrate treatment in the porcine alveolar macrophages. Front. Immunol..

[24] Zhang Q., Cui C., Zhang S., Deng X., Cai X., Wang G. (2022). Tylvalosin Tartrate Improves the Health Status of Swine Herds during Immunization with Porcine Reproductive and Respiratory Syndrome Virus-Inactivated Vaccine. Vet. Sci..

[25] Zhang H.L., Zhang W.L., Xiang L.R., Leng C.L., Tian Z.J., Tang Y.D., Cai X.H. (2018). Emergence of novel porcine reproductive and respiratory syndrome viruses (ORF5 RFLP 1-7-4 viruses) in China. Vet. Microbiol..

[26] Zhao K., Ye C., Chang X.B., Jiang C.G., Wang S.J., Cai X.H., Tong G.Z., Tian Z.J., Shi M., An T.Q. (2015). Importation and Recombination Are Responsible for the Latest Emergence of Highly Pathogenic Porcine Reproductive and Respiratory Syndrome Virus in China. J. Virol..

[27] Zhou L., Wang Z., Ding Y., Ge X., Guo X., Yang H. (2015). NADC30-like Strain of Porcine Reproductive and Respiratory Syndrome Virus, China. Emerg. Infect. Dis..

[28] Tang X., Wang C., Sun W., Wu W., Sun S., Wan J., Zhu G., Ma N., Ma X., Xu R. (2023). Evaluating anti-viral effect of Tylvalosin tartrate on porcine reproductive and respiratory syndrome virus and analyzing the related gene regulation by transcriptomics. Virol. J..

[29] Zheng S., An H., Xu F., Meng F., Cai X., Tian Z., Peng J., Deng X., Shang Y., Wang G. (2024). Tylvalosin tartrate inhibits the replication stage of the porcine reproductive and respiratory syndrome virus life cycle. Microb. Pathog..

[30] Cui M., Qiu M., Yang S., Qiu Y., Qi W., Lin H., Sun Z., Zheng W., Zhu J., Chen N. (2025). The replication efficacy of NADC34-like porcine reproductive and respiratory syndrome virus 2 is not directly associated with the pathogenicity. Vet. Microbiol..

[31] Yu P.W., Fu P.F., Zeng L., Qi Y.L., Li X.Q., Wang Q., Yang G.Y., Li H.W., Wang J., Chu B.B. (2022). EGCG Restricts PRRSV Proliferation by Disturbing Lipid Metabolism. Microbiol. Spectr..

[32] Du T., Shi Y., Xiao S., Li N., Zhao Q., Zhang A., Nan Y., Mu Y., Sun Y., Wu C. (2017). Curcumin is a promising inhibitor of genotype 2 porcine reproductive and respiratory syndrome virus infection. BMC Vet. Res..

[33] Zeng S., Meng X., Huang Q., Lei N., Zeng L., Jiang X., Guo X. (2019). Spiramycin and azithromycin, safe for administration to children, exert antiviral activity against enterovirus A71 in vitro and in vivo. Int. J. Antimicrob. Agents.

