# Supplementary figures and images for: Breaking the PRRSV-2 Life Cycle in Porcine Alveolar Macrophages: Tylvalosin’s Multi-Stage Inhibition
Source: Vet Sci. 2025 Apr 9;12(4):348. doi: 10.3390/vetsci12040348 (PMC12031314; doi:10.3390/vetsci12040348)

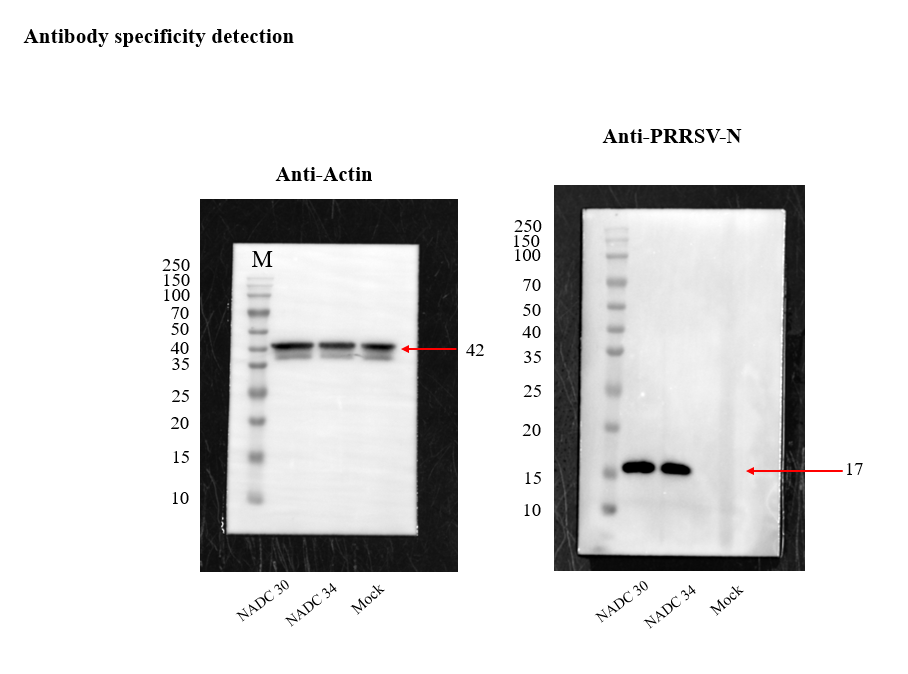

Supplement: Supplementary file 1 [file vetsci-12-00348-s001.zip › vetsci-3525268-supplementary/Original images/Antibody-specific results/a13a50fd84913fb792f7de60e85e941.png]

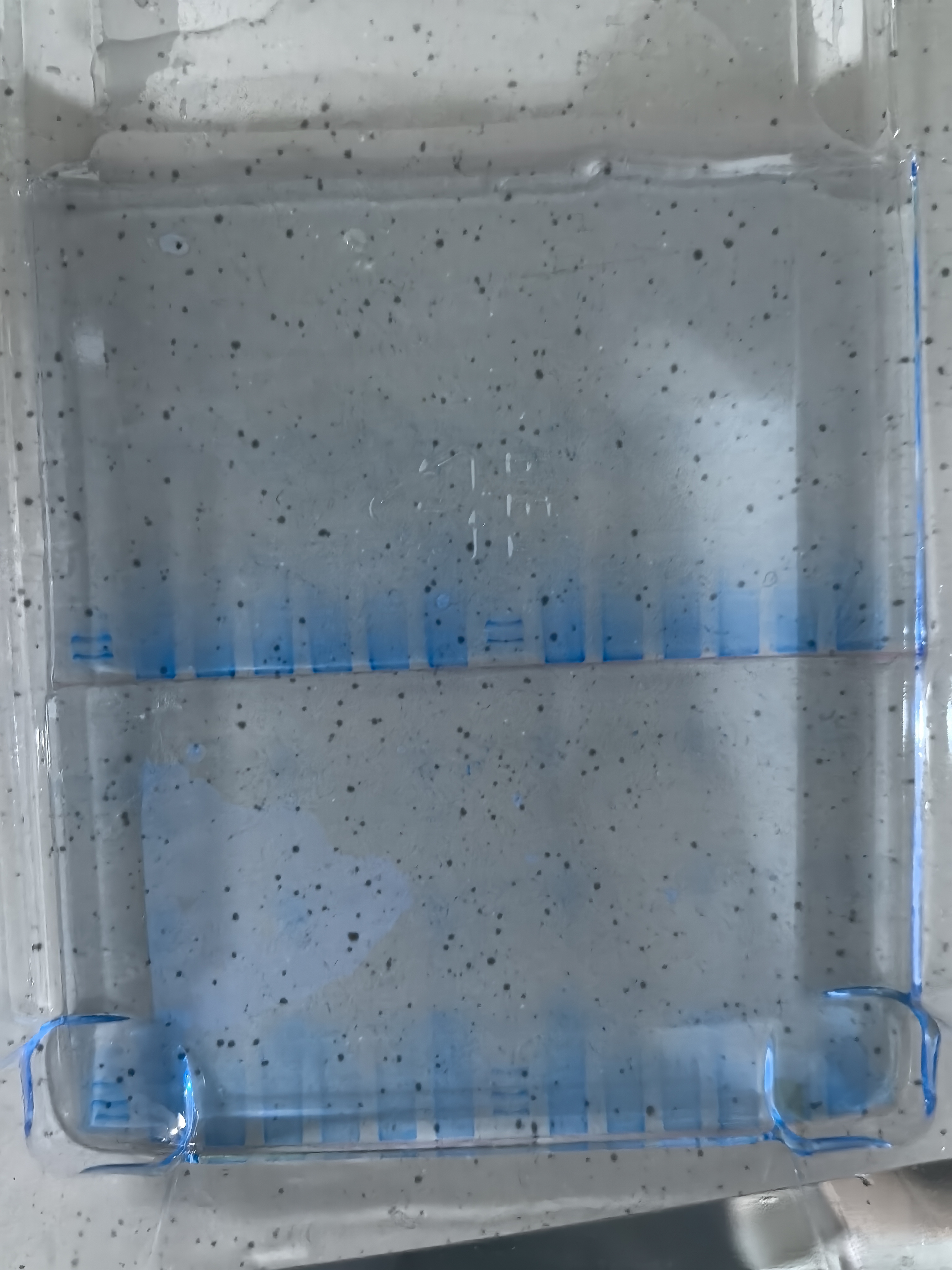

Supplement: Supplementary file 1 [file vetsci-12-00348-s001.zip › vetsci-3525268-supplementary/Original images/Figure 3/The blot images/Figure3 A.B.C UP.jpg]

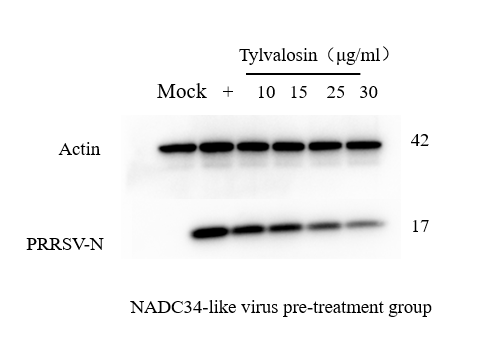

Supplement: Supplementary file 1 [file vetsci-12-00348-s001.zip › vetsci-3525268-supplementary/Original images/Figure 3/The gel images/Figure 3 A down/Image annotations 1.png]

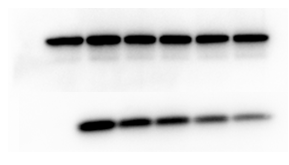

Supplement: Supplementary file 1 [file vetsci-12-00348-s001.zip › vetsci-3525268-supplementary/Original images/Figure 3/The gel images/Figure 3 A down/total protein.png]

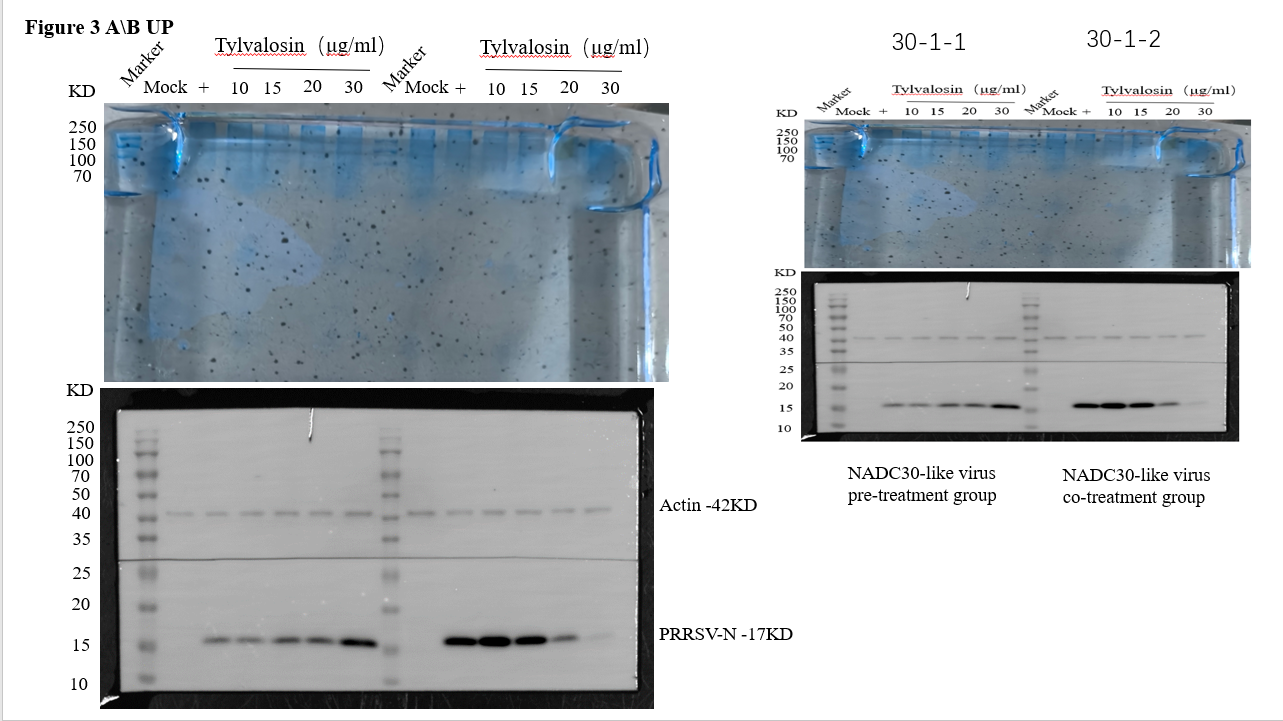

Supplement: Supplementary file 1 [file vetsci-12-00348-s001.zip › vetsci-3525268-supplementary/Original images/Figure 3/The gel images/Figure 3 A.B UP/Image annotations 1.png]

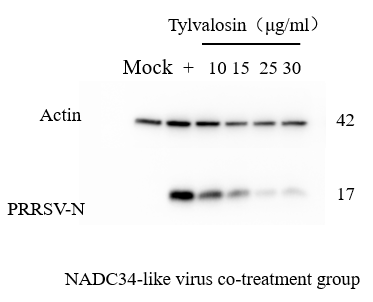

Supplement: Supplementary file 1 [file vetsci-12-00348-s001.zip › vetsci-3525268-supplementary/Original images/Figure 3/The gel images/Figure 3 B down/Image annotations 2.png]

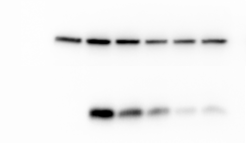

Supplement: Supplementary file 1 [file vetsci-12-00348-s001.zip › vetsci-3525268-supplementary/Original images/Figure 3/The gel images/Figure 3 B down/total protein.png]

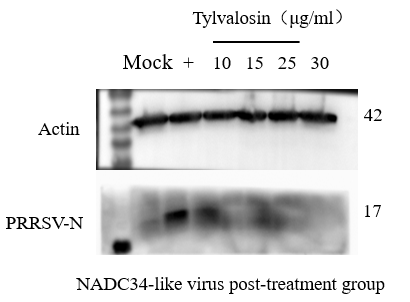

Supplement: Supplementary file 1 [file vetsci-12-00348-s001.zip › vetsci-3525268-supplementary/Original images/Figure 3/The gel images/Figure 3 C down/Image annotations 3.png]

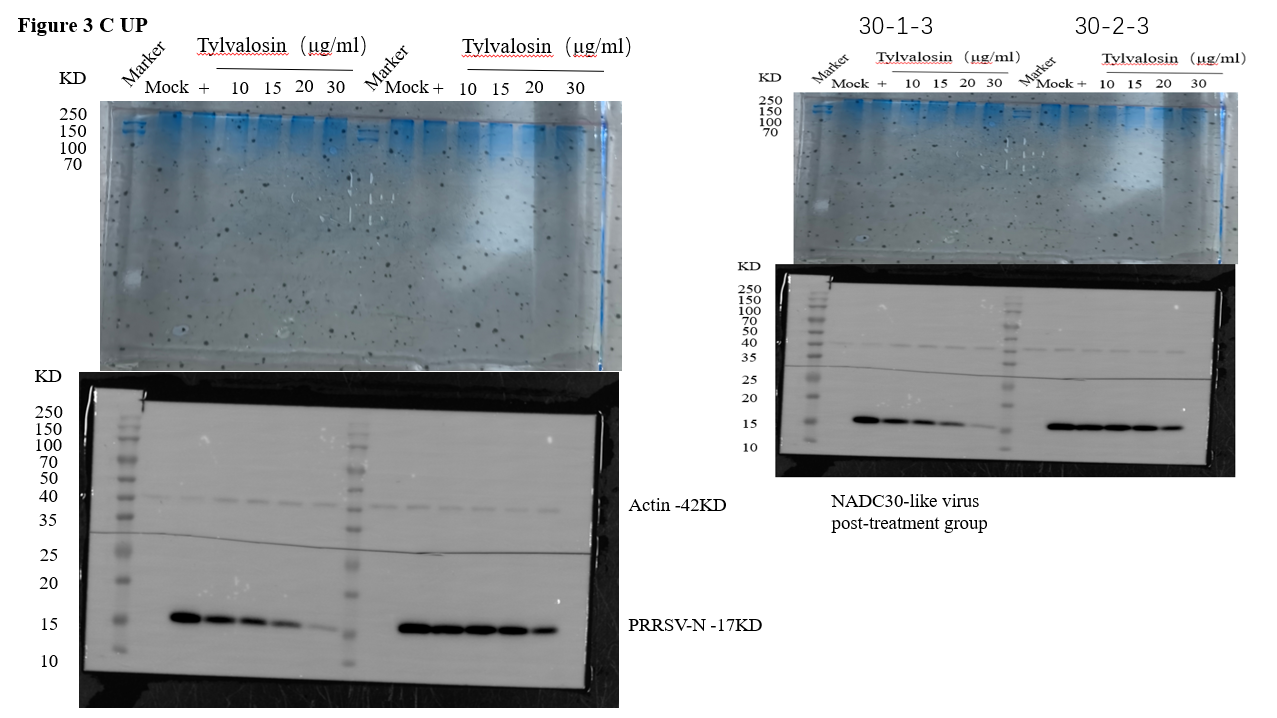

Supplement: Supplementary file 1 [file vetsci-12-00348-s001.zip › vetsci-3525268-supplementary/Original images/Figure 3/The gel images/Figure 3 C UP/Image annotations 2.png]

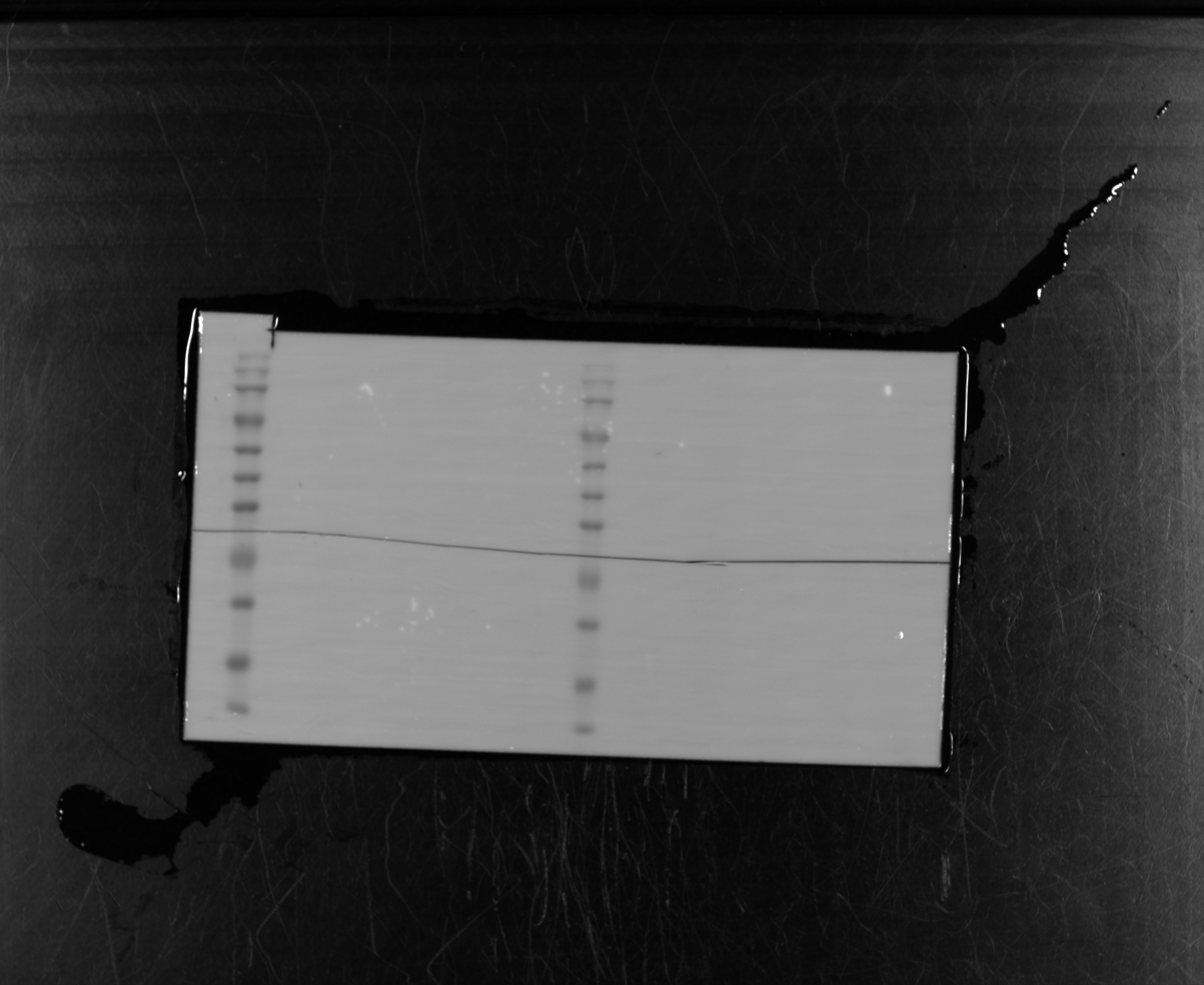

Supplement: Supplementary file 1 [file vetsci-12-00348-s001.zip › vetsci-3525268-supplementary/Original images/Figure 3/The gel images/Figure 3 C UP/Total Marker.Tif]

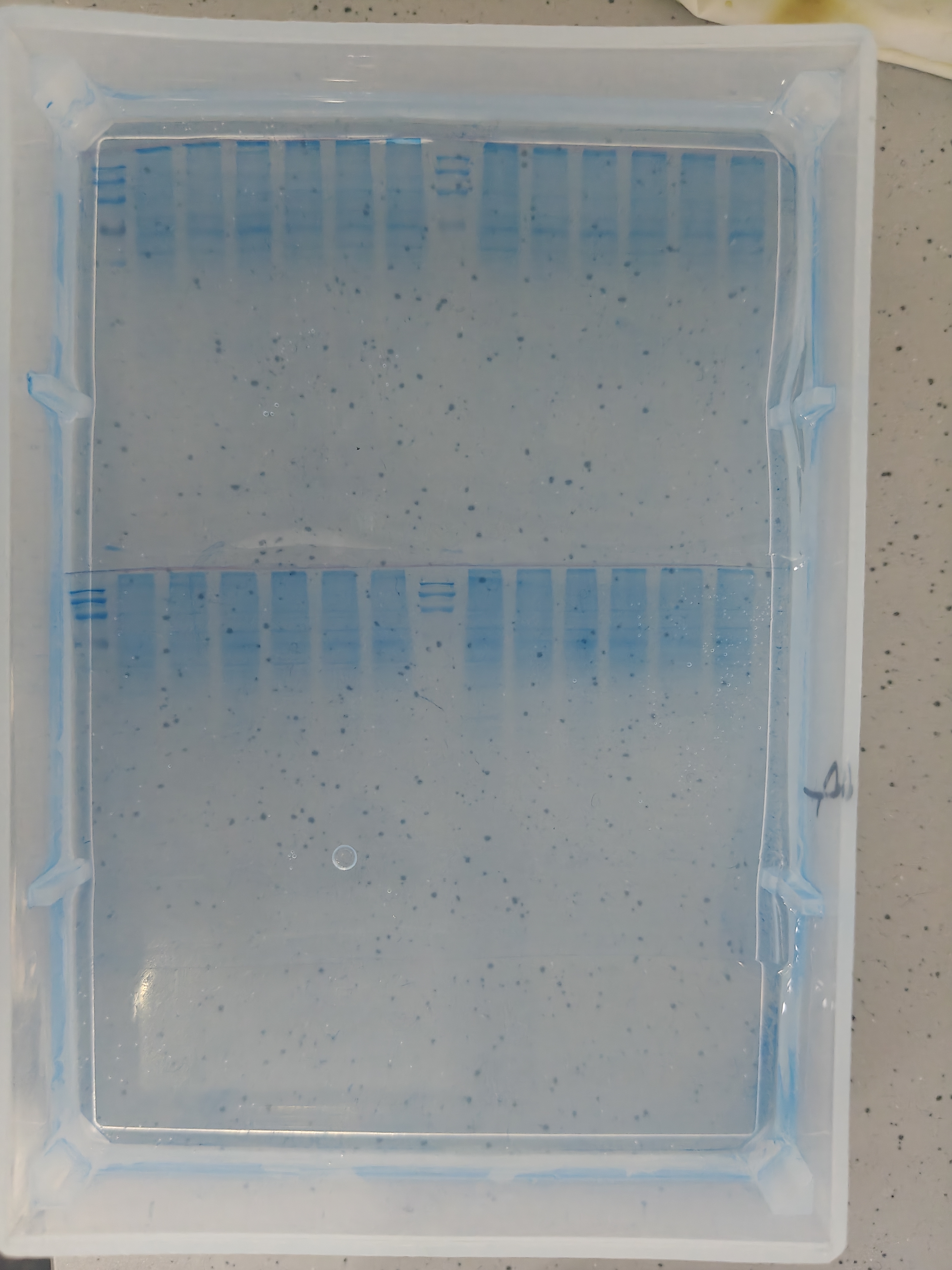

Supplement: Supplementary file 1 [file vetsci-12-00348-s001.zip › vetsci-3525268-supplementary/Original images/Figure 4/The bolt images/Figure 4 A.B.C.D left.jpg]

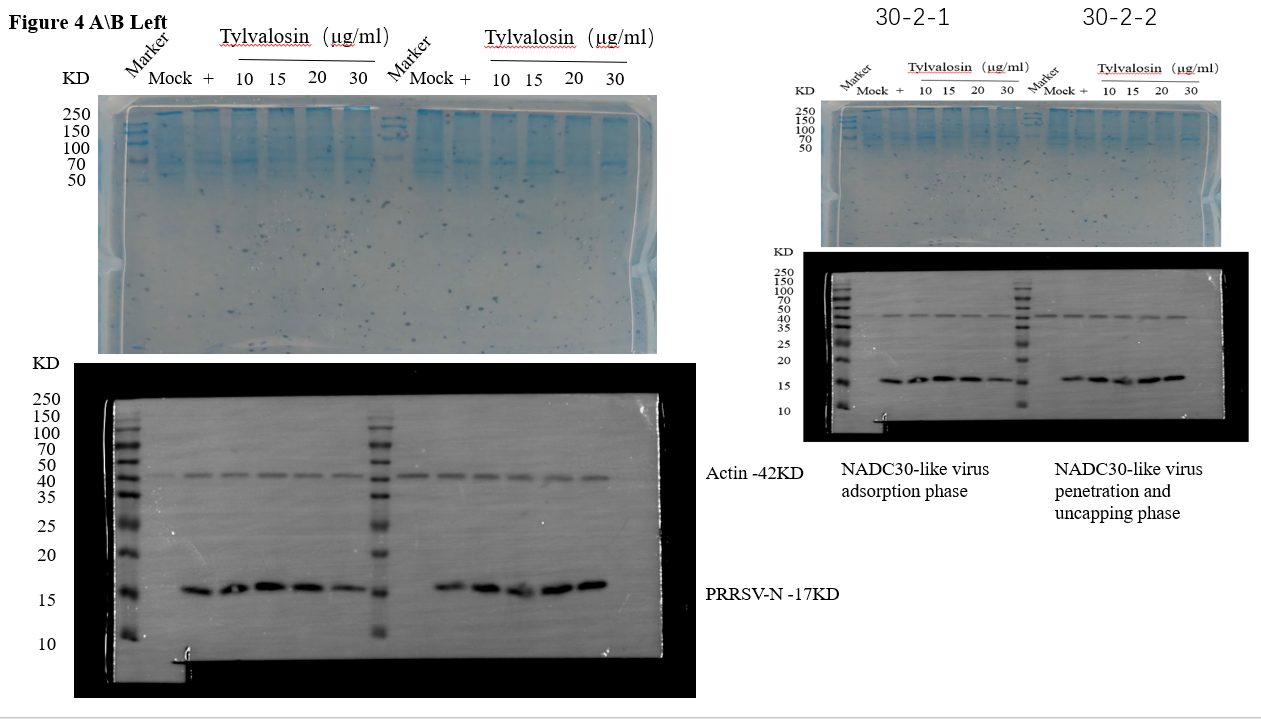

Supplement: Supplementary file 1 [file vetsci-12-00348-s001.zip › vetsci-3525268-supplementary/Original images/Figure 4/The gel images/Figure 4 A.B left/Image annotations 1.png]

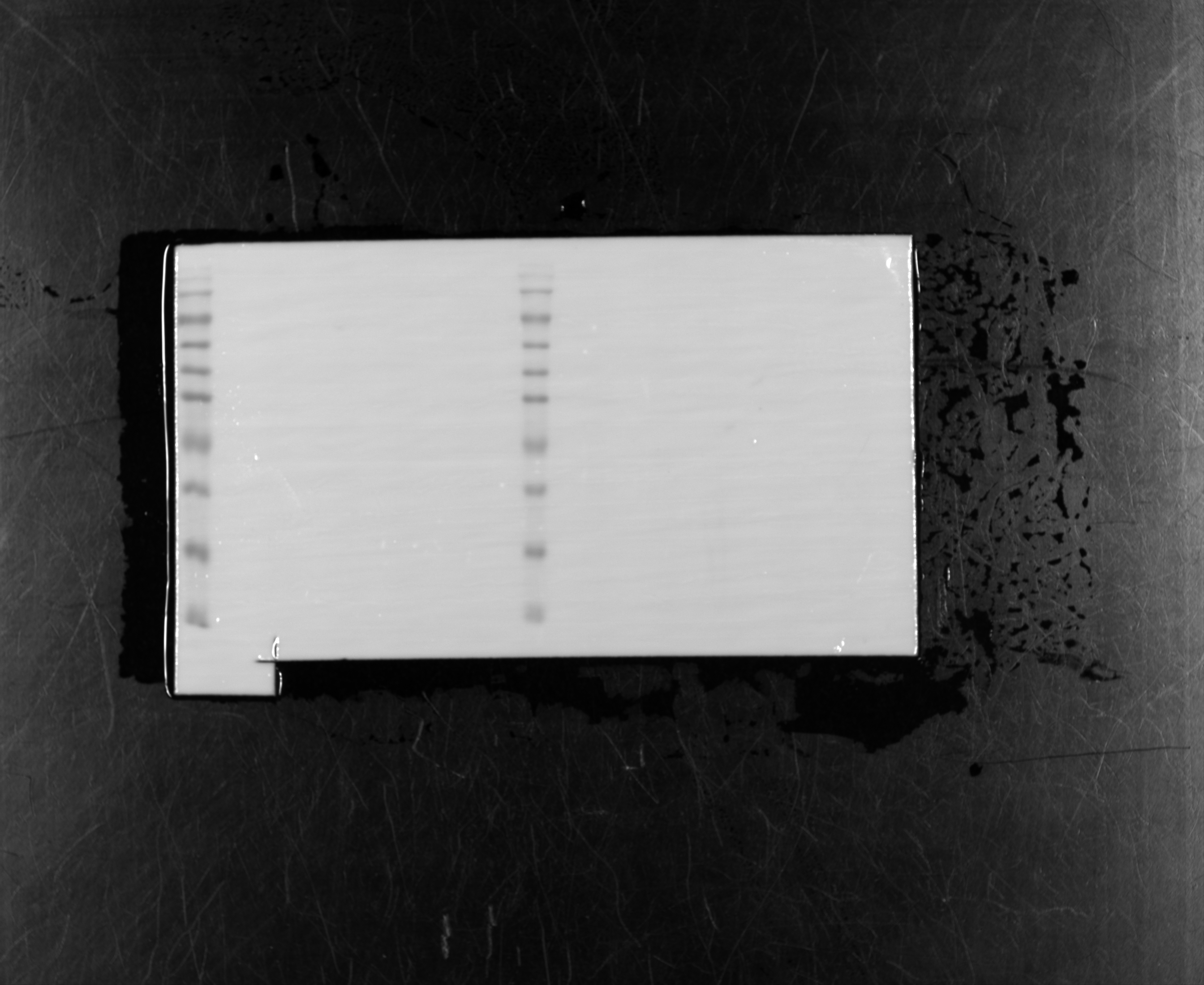

Supplement: Supplementary file 1 [file vetsci-12-00348-s001.zip › vetsci-3525268-supplementary/Original images/Figure 4/The gel images/Figure 4 A.B left/total marker.Tif]

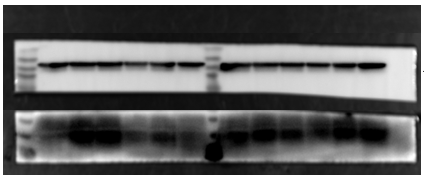

Supplement: Supplementary file 1 [file vetsci-12-00348-s001.zip › vetsci-3525268-supplementary/Original images/Figure 4/The gel images/Figure 4 A.B.C.D right/A. total merge.png]

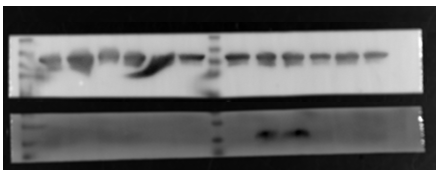

Supplement: Supplementary file 1 [file vetsci-12-00348-s001.zip › vetsci-3525268-supplementary/Original images/Figure 4/The gel images/Figure 4 A.B.C.D right/B. total merge.png]

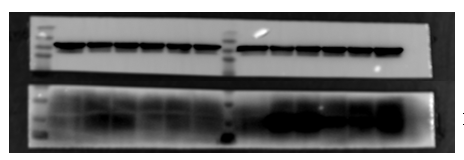

Supplement: Supplementary file 1 [file vetsci-12-00348-s001.zip › vetsci-3525268-supplementary/Original images/Figure 4/The gel images/Figure 4 A.B.C.D right/C. Total merge.png]

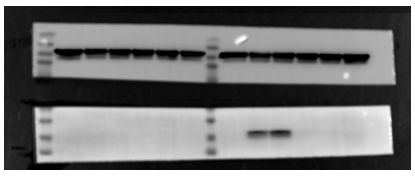

Supplement: Supplementary file 1 [file vetsci-12-00348-s001.zip › vetsci-3525268-supplementary/Original images/Figure 4/The gel images/Figure 4 A.B.C.D right/D. total merge.png]

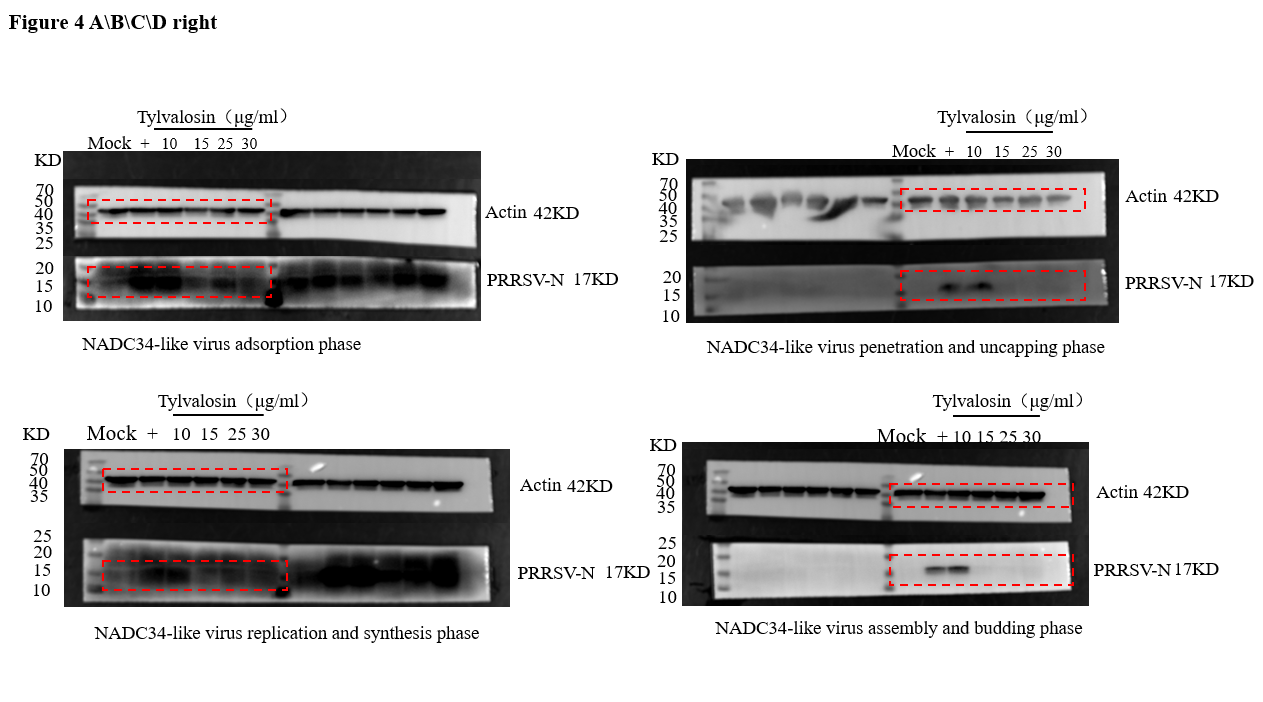

Supplement: Supplementary file 1 [file vetsci-12-00348-s001.zip › vetsci-3525268-supplementary/Original images/Figure 4/The gel images/Figure 4 A.B.C.D right/Image annotations 1.png]

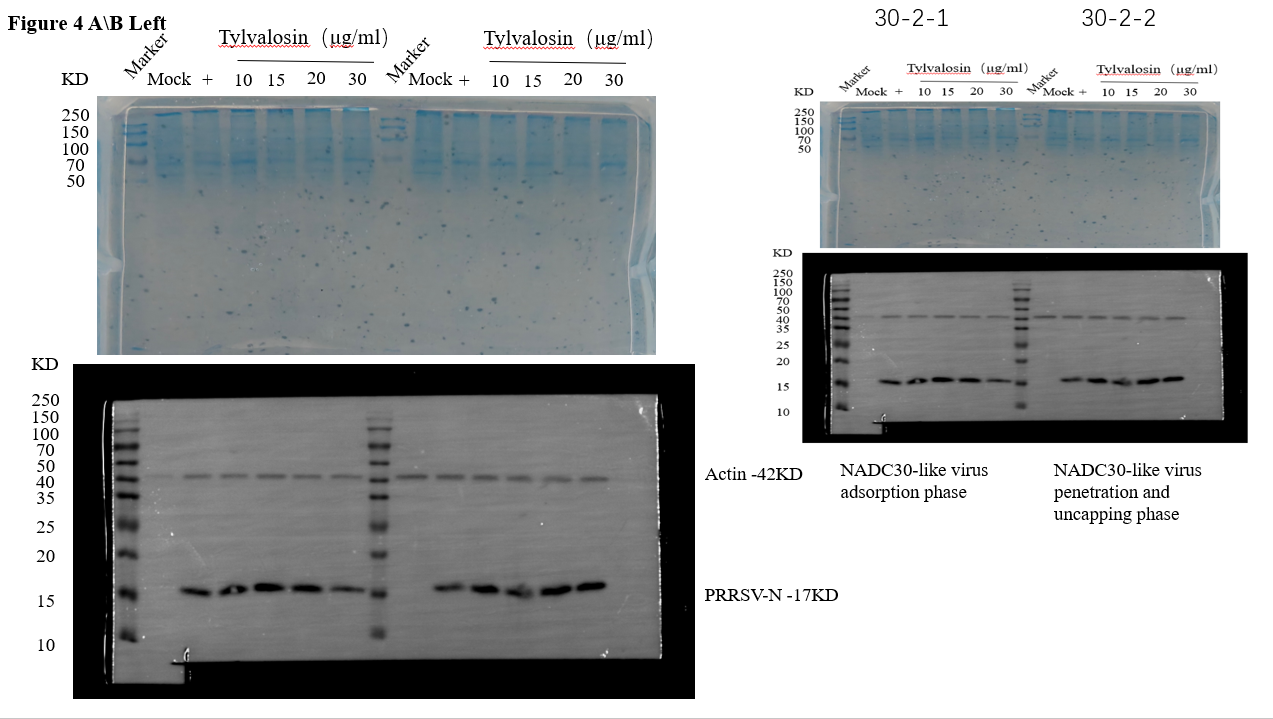

Supplement: Supplementary file 1 [file vetsci-12-00348-s001.zip › vetsci-3525268-supplementary/Original images/Figure 4/The gel images/Figure 4 C.D left/Image annotations 2.png]

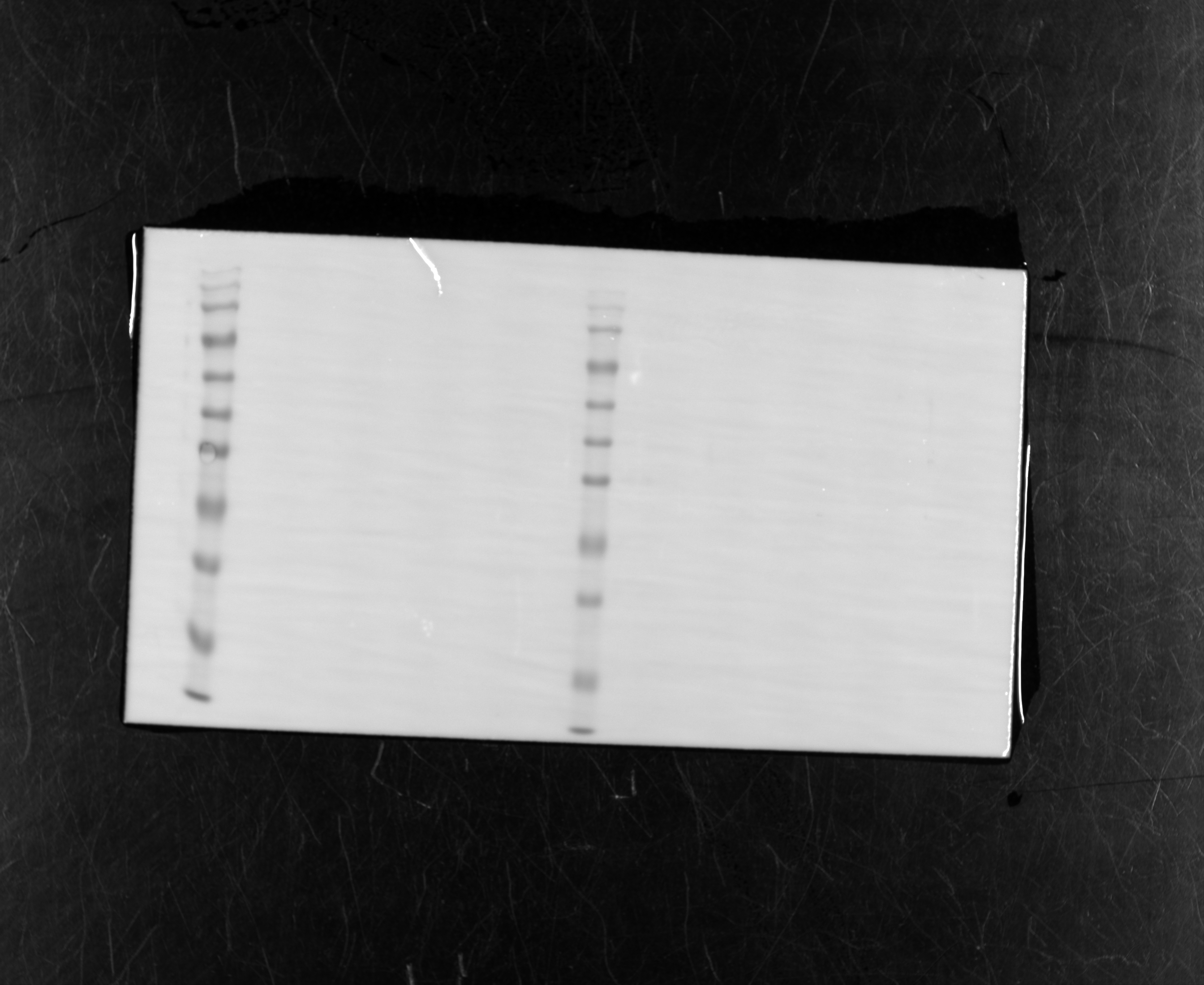

Supplement: Supplementary file 1 [file vetsci-12-00348-s001.zip › vetsci-3525268-supplementary/Original images/Figure 4/The gel images/Figure 4 C.D left/total marker.Tif]
